# Use of Simulation Based on an Electronic Health Records Environment to Evaluate the Structure and Accuracy of Notes Generated by Medical Scribes: Proof-of-Concept Study

**DOI:** 10.2196/medinform.7883

**Published:** 2017-09-20

**Authors:** Robert Pranaat, Vishnu Mohan, Megan O'Reilly, Maxwell Hirsh, Karess McGrath, Gretchen Scholl, Deborah Woodcock, Jeffrey A Gold

**Affiliations:** ^1^ Medical Informatics Oregon Health & Sciences University Portland, OR United States; ^2^ Obstetrics and Gynecology Oregon Health & Sciences University Portland, OR United States; ^3^ School of Medicine Oregon Health & Sciences University Portland, OR United States; ^4^ Pulmonary Critical Care Oregon Health & Sciences University Portland, OR United States

**Keywords:** simulation training, documentation, electronic health record

## Abstract

**Background:**

The increasing adoption of electronic health records (EHRs) has been associated with a number of unintended negative consequences with provider efficiency and job satisfaction. To address this, there has been a dramatic increase in the use of medical scribes to perform many of the required EHR functions. Despite this rapid growth, little has been published on the training or assessment tools to appraise the safety and efficacy of scribe-related EHR activities. Given the number of reports documenting that other professional groups suffer from a number of performance errors in EHR interface and data gathering, scribes likely face similar challenges. This highlights the need for new assessment tools for medical scribes.

**Objective:**

The objective of this study was to develop a virtual video-based simulation to demonstrate and quantify the variability and accuracy of scribes’ transcribed notes in the EHR.

**Methods:**

From a pool of 8 scribes in one department, a total of 5 female scribes, intent on pursuing careers in health care, with at least 6 months of experience were recruited for our simulation study. We created three simulated patient-provider scenarios. Each scenario contained a corresponding medical record in our simulation instance of our EHR. For each scenario, we video-recorded a standardized patient-provider encounter. Five scribes with at least 6 months of experience both with our EHR and in the specialty of the simulated cases were recruited. Each scribe watched the simulated encounter and transcribed notes into a simulated EHR environment. Transcribed notes were evaluated for interscribe variability and compared with a gold standard for accuracy.

**Results:**

All scribes completed all simulated cases. There was significant interscribe variability in note structure and content. Overall, only 26% of all data elements were unique to the scribe writing them. The term *data element* was used to define the individual pieces of data that scribes perceived from the simulation. Note length was determined by counting the number of words varied by 31%, 37%, and 57% between longest and shortest note between the three cases, and word economy ranged between 23% and 71%. Overall, there was a wide inter- and intrascribe variation in accuracy for each section of the notes with ranges from 50% to 76%, resulting in an overall positive predictive value for each note between 38% and 81%.

**Conclusions:**

We created a high-fidelity, video-based EHR simulation, capable of assessing multiple performance indicators in medical scribes. In this cohort, we demonstrate significant variability both in terms of structure and accuracy in clinical documentation. This form of simulation can provide a valuable tool for future development of scribe curriculum and assessment of competency.

## Introduction

The electronic health record (EHR) is a vital tool in the delivery of clinical care. The EHR adoption rates have grown rapidly largely because of government programs such as the Health Technology for Economic and Clinical Health (HITECH) Act of 2009 [1]. However, physician dissatisfaction with EHRs remains high, a phenomenon probably linked to the perception that EHRs do not improve efficiency (42%), do not decrease workload (72%), have increased total operating costs (54%), and have yet to overcome operating challenges (43%) [2].

One key factor that contributes to the dissatisfaction is the paradigm of “information chaos” resulting from EHR use that can lead to impaired situational awareness and increased mental workload [3]. To amplify this paradigm, a number of studies conducted by our group and others have suggested that providers across multiple professions have difficulty in using the EHR as manifested by issues with data finding, recognition of patient safety issues, and impairment in clinical decision making [4,5]. Additionally, recent studies revealed that problems associated with clinicians’ selective data gathering or selective data interpretation can lead to increased patient harm, a phenomenon that has also been identified and replicated in simulation exercises [6,7]. These issues are not just isolated to physicians: recent work from our group has suggested that the phenomenon affects nurses and pharmacists at all levels of training, implying a global problem related to human EHR interfaces [5,8].

Growing concerns with EHR usability and efficiency have been mirrored by concomitant increased utilization of medical scribes. To alleviate challenges associated with EHR data entry, physicians have increasingly incorporated scribes into clinic and hospital workflows. Though studies lauding their potential benefits have been present for nearly 30 years, recently the scribe workforce has demonstrated a significant and rapid growth; there were approximately 10,000 scribes working in 2014 with a projection of 20,000 scribes in the workforce by 2016 [9,10]. However, whereas the number of scribes has increased dramatically, there still exists no standardized approach for training and assessing scribes. Before being embedded within a practice, scribes have varied levels of clinical exposure and disparate degrees of training varying from formal EHR training by employers or scribe organizations to Web-based courses by commercial scribe solution organizations to ad hoc training conducted by clinicians to no training at all. This often creates an interesting paradox: most physicians feel that their own training with the EHR is inadequate and their need for utilizing scribes arises from their inability to use the EHR in a safe and efficient manner [2,11,12]. Yet, these physicians may then be responsible for training and assessing scribes who have had often little to no direct health care experience themselves.

Scribes who use the EHR may find the complex interface and usability constraints of the EHR potentially even more challenging than physicians do because they lack clinical learning and EHR-specific workflow training. In essence, this paradigm adds another layer of physician responsibility but does not eliminate the errors inherent with poor EHR use.

These issues are further magnified by the fact that scribes do not necessarily just engage in data entry activities during the clinical encounter but may also have a variable and expanded role at the discretion of the provider they are scribing for [13,14]. Currently, the only defined regulatory guidance for scribe use comes from The Joint Commission, which deems that medical scribes are to “chart at the direction of their provider” and should not place orders. Furthermore, physicians are required by the Joint Commission to authenticate, or attest to, all notes written by scribes [14].

To ensure that standardized activities are accomplished, scribes require appropriate training that directly links their learning needs with measured outcomes. This can be accomplished through training regimens that evaluate individual competencies pertinent to accurate EHR documentation. Training should maintain Health Insurance Portability and Accountability Act (HIPAA) compliance and ensure patient safety. Given the relationship between communication errors and patient safety [15], scribes’ role in EHR documentation stands to benefit from training that does not endanger patient well-being.

On the basis of these concerns, it is imperative that methodology exists to ensure that scribes can be effectively trained and their competency assessed for safe and effective use of EHR in the appropriate clinical settings. Simulation has been a means of evaluating complicated systems, while posing no risk to patients, and providing high-fidelity standardized subject experiences [4,5]. Recently, we demonstrated that EHR-based simulation could be used to assess the creation and accuracy of both intern progress notes and admission history and physicals [16,17]. Given that high-fidelity simulation is effective with regard to facilitating improved EHR use for multiple clinical professions such as physicians, nurses, and pharmacists, it seems logical that similar techniques would also be effective for scribes, whose role as EHR documentation experts essentially replaces these same skills by physicians. Therefore, our hypothesis is that through the use of high-fidelity simulated provider-patient encounters and integrated EHR, it is possible to assess scribes’ EHR use in similar fashion.

## Methods

The study was approved by the institutional review board of the Oregon Health & Science University. All data were deidentified and stored securely.

### Simulation Creation and Materials

Three Obstetrics-Gynecology (Ob-Gyn) scenarios were created by a clinical subject matter expert (Ob-Gyn attending physician) to represent standard ambulatory encounters. We created a replica of each clinical case in our simulation instance of EpicCare (Epic Systems) using techniques we have described in previous publications [4,18]. Briefly, the EHR instance utilized for simulation activities is created from a “clone” of the clinical system, maintaining all user customizations, shortcuts, and macros. The instance contains only patient charts representing the simulation; it does not contain protected health information of real patients in our health care system. Given the need for any simulation-based training exercise to be both scalable and accessible from a variety of clinical environments, we decided to use a virtual video-based simulation. For each scenario, we video-recorded a standardized patient-provider encounter, with medical personnel serving in roles of both physician and patient. Once recorded, each video was cropped and edited to ensure adequate audio and video quality. On the basis of the script of each scenario, a “gold-standard” note was created for each case to allow for assessment of accuracy of content of individual scribe notes.

### Subject Recruitment and Characteristics

A list of all medical scribes was collected from the Scribe Program Supervisor of the OHSU medical scribing program. Medical scribes working at the OHSU Center for Women’s Health (CWH) were selected because they represented the largest proportion of all medical scribes working at OHSU. They were approached via email, phone texts, and phone calls to arrange simulation participation times. All scribes had a minimum of 1 year of scribe experience and minimum 6 months of experience scribing for CWH before study participation.

### Simulation Procedure

In order for the simulations to accurately replicate scribes’ work environment in real-world settings, the activity was conducted at the CWH, OHSU. For each simulated case, subjects were instructed to (1) familiarize themselves with each simulated patient chart before beginning the simulated physician-patient video, and (2) perform scribe activities in simulation just as they would during a real physician-patient interaction.. Simulations were performed in patient exam rooms at the CWH, OHSU that replicated real-world conditions accurately. Videos were displayed from a laptop computer on the exam table. Scribes used dedicated exam-room computers. The standardized narrative was read aloud to each scribe. Each simulation lasted between 6 and 18 min and scribes performed all three cases, in the same order.

### Data Collection

Scribe- and physician-created notes were transferred from the Epic simulation environment into Pages (Apple Inc). Screenshots were taken of the Encounter, Labs, and Imaging tabs of Chart Review to determine whether the orders were pended. The gold-standard note was transferred from the Epic simulation environment into Pages in the same manner.

### Data Analysis

Scribe notes were evaluated for note length, word economy, data elements, copy and paste blocks, pended orders, and attestations. These structural elements were compared with each other to determine interscribe variability. Structural elements were also compared with our gold-standard note to determine accuracy and positive predictive value (PPV). PPV was defined as the ratio of scribe’s data elements also found in the gold-standard note to all those data elements included by the scribe. Data elements were defined as the individual positive and negative facts created by the scribe or gold standard from each of the patient-physician videos and provided resources. Data elements represented the interpretation of the scribe and the gold standard with respect to what was verbalized and performed during the encounter. Data elements were tabulated by note section, subjective, objective, or assessment and plan. The presence of copy and pasted blocks was determined using Plagiarism Checker X (Plagiarism Checker X, LLC), a plagiarism detection software package. Word economy was defined as the number of words required to create 1 data element or the number of words divided by data elements. Attestations were considered present if the medical-scribe included a statement at the end of their note signifying that they were a scribe working on behalf of the physician-provider.

## Results

**Figure 1:**
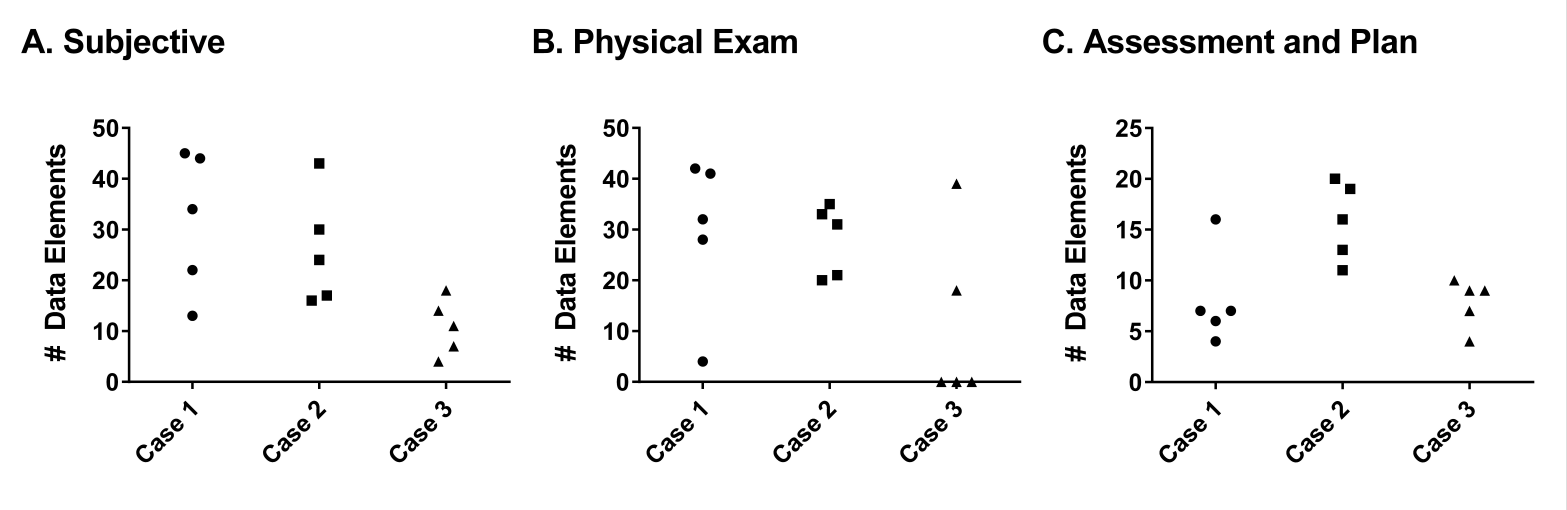
Distribution of data elements. Each of the 5 scribes completed 3 separate simulation exercises. The absolute number of data elements for each section of the note was tabulated for each individual scribe. Subjective (Panel A), Physical exam (Panel B), and Assessment and plan (Panel C).

We first wanted to determine the general structure and interscribe variability determined by data elements, note length, word economy, pended orders, attestations, and the specific structure of each note section. A total of 150, 183, and 118 unique data elements were found in case 1, case 2, and case 3, respectively (Figure 1). Upon examining interscribe variability in elements, there was a 2- to 4-fold range in the number of data elements present for each range of data elements among the 5 scribes.

**Figure 2:**
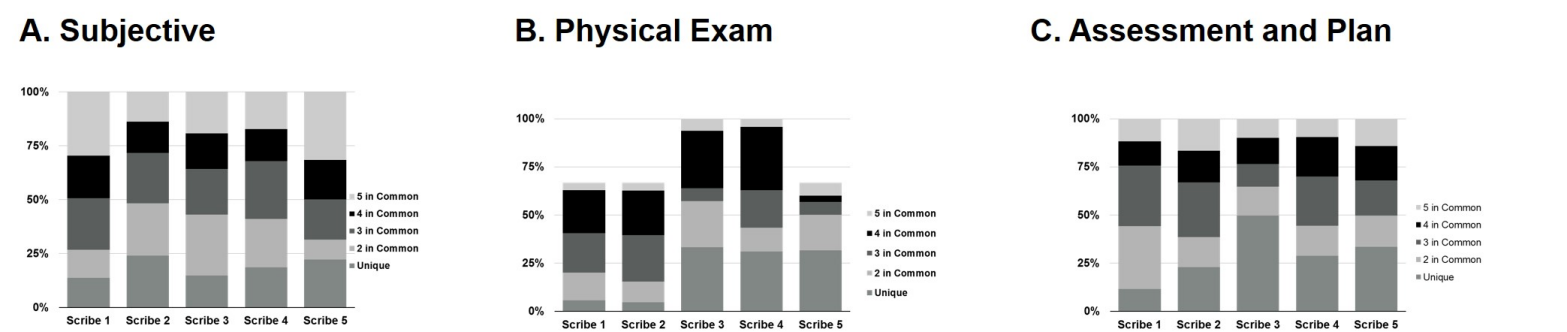
Interscribe commonality in data elements. Each of the 5 scribes completed 3 separate simulation exercises. For each section of the note, Subjective (Panel A), Physical exam (Panel B), and Assessment and plan (Panel C), the fraction of data elements for each scribe in common among the other scribes for all three cases is presented.

We next sought to determine the commonality of data elements between scribes. For each scribe, for a given element, we determined what fraction of the total cohort of scribes documented this element in their note for and individual case. Data from all three cases were then pooled for analysis. We further subdivided the analysis to the three main sections: Subjective, Physical exam, and Assessment and plan (Figure 2). Of interest, in the subjective section, less than 25% of data elements in an individual scribes’ note were represented in all 5 of the notes, whereas almost 20% were unique to the individual scribe. Further, when analyzing the physical exam, scribe 3 and 4 documented elements that were not present in the simulation for case 3, explaining the inability of notes from the remaining scribes to have any elements present in 100% of the cohorts’ note. Overall, 26% of all scribe-created data elements were unique to individual scribes, whereas 17% of all data elements created by scribes received complete agreement.

**Figure 3:**
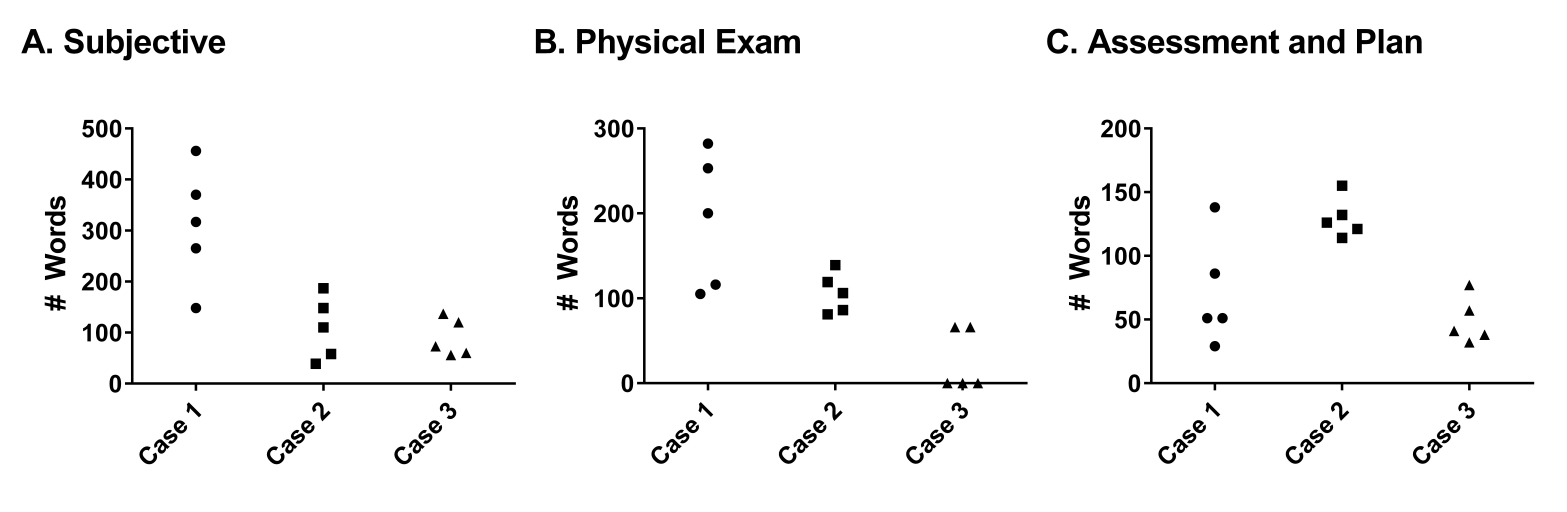
Distribution of Word Count. Five scribes each completed 3 separate simulation exercises. The absolute number of words for each section of the note was tabulated for each individual scribe. Subjective (Panel A), Physical Exam (Panel B), and Assessment and Plan (Panel C).

These differences in note elements were associated with significant variability in global note structure and content. There was almost an 87-fold difference in note length in case 1 between the high and low, 55-fold difference in case 2, and 115-fold difference in case 3. Of note, variance was observed across all structural domains of the note (Figure 3). In case 1, the shortest note was 37% (293/794) of the longest note, in case 2, it was 57% (251/440), and in case 3, the shortest note represented 31% (94/302) of the length of the longest note.

Finally, we wished to determine differences in the general structure of scribes’ note with that of the gold-standard note. Errors of omission were demonstrated by calculating for accuracy, that is, the frequency by which scribes included all the data elements that were found in the gold-standard note. Similarly, errors of commission were demonstrated through the use of PPV, whereby we were able to calculate how often scribes in our study included information that was not present, and therefore assumed to be inaccurate, in the gold-standard note. Individual scribe accuracy ranged from 50% to 76%, whereas the accuracy of subjective, objective, and assessment and plan was 72%, 60%, and 56%, respectively. For individual scribes the PPV ranged from 38% to 81%. When scribe notes were averaged, the PPV of subjective, objective, and assessment and plan was 54%, 52%, and 69%, respectively (Table 1).

**Table 1:** Accuracy and Positive Predictive Value (PPV) for each simulated case by structural element.

Note section		Case #1										Case #2										Case #3									
			1		2		3		4		5		1		2		3		4		5		1		2		3		4		5
**True Positive**																															
	Subjective		16		34		31		24		10		23		33		16		15		12		4		10		9		8		8
	PE		14		15		4		1		0		12		13		4		8		6										
	A&P		6		7		3		4		2		13		9		16		15		11		2		4		2		2		1
**False Positive**																															
	Subjective		6		11		13		10		3		7		12		15		9		8		0		1		9		6		1
	PE		3		2		4		3		4		1		3		4		3		4										
	A&P		3		2		14		0		4		3		2		3		5		2		7		6		5		7		3
**False Negative**																															
	Subjective		34		16		19		26		40		28		18		35		36		39		2		2		2		2		2
	PE		2		1		12		15		16		2		1		10		6		8										
	A&P		2		1		2		3		3		4		4		3		4		4		1		1		1		1		1
**Accuracy**																															
	Subjective		0.73		0.76		0.7		0.71		0.77		0.77		0.73		0.52		0.63		0.6		1		0.91		0.5		0.57		0.86
	PE		0.82		0.88		0.5		0.25		0		0.92		0.81		0.5		0.73		0.6										
	A&P		0.67		0.78		0.18		1		0.33		0.81		0.82		0.84		0.75		0.85		0.22		0.4		0.29		0.22		0.25
**PPV**																															
	Subjective		0.32		0.68		0.62		0.48		0.2		0.45		0.65		0.31		0.29		0.24		0.67		0.83		0.82		0.8		0.75
	PE		0.88		0.94		0.25		0.06		0		0.86		0.93		0.29		0.57		0.43										
	A&P		0.75		0.88		0.6		0.57		0.4		0.76		0.69		0.84		0.79		0.73		0.67		0.8		0.67		0.67		0.5

## Discussion

### Principal Findings

In this study, we created a novel virtual simulation to specifically assess scribe use and function. The use of a standardized video encounter carries the distinct advantage of untethering the simulation from a traditional simulation center, thereby improving accessibility of the training activity to multiple clinical environments. This represents a more scalable alternative, given how scribes are already reported to work in a variety of clinical environments and are deeply embedded in community clinics, many of which may not have access to traditional simulation. In addition, the use of a standardized video ensures consistency of the delivery of content, allowing for direct comparison of work-product between scribes and across practices.

With the standardization of the delivery of content and inclusion of the EHR as an integral part of the simulation activity, we were able to allow direct interscribe comparisons between notes, which revealed significant variability in note structure and length. There is a lack of clarity with respect to the extent of experience medical scribes require to attain any particular level of competency. Despite the fact that all of the scribes had at least 1 year of experience both in the specialty and with the EHR, there was almost a 3-fold difference in note length. Even more interesting was the difference in actual “note” elements between scribes. This is consistent with findings from studies showing discrepancies between physicians in the content and quality of documentation in notes [19,20]. Thus, whereas this phenomenon is most likely not unique to scribes, it does imply that scribes may face the same issues that are found among other clinicians.

Although the simulation provides the basis to assess differences in note structure, we were also able to create a methodology to look at note content. We found evidence of errors of commission (incorrect data) and omission (missing data) by comparing the data elements found in notes written by scribes versus the notes written by an expert clinician. Notably, there was a paucity of overlap in content between the notes, with less than 40% of the documented plan items and diagnoses being common across the scribes. This is consistent with the observation that there is wide variability in the content of resident-physician-generated progress notes, where the primary author of the note (the resident) was also responsible for acquisition of the primary data and synthesizing that information into medical decision making [20]. This study suggests that similar issues may arise purely in the process of how our subjects communicate as members of an interprofessional team. However, this study does not delineate whether the differences observed are because of the individual scribe workflows, scribe deficits in medical knowledge, issues related to scribe training, or lacunae in scribe-physician communication. The use of a controlled simulated case may also explain the differences between our results and a recent study looking at actual scribe-generated notes in a practice setting [21]. In that study, scribe-generated notes for diabetes encounters, with medical assistants serving as scribes, created equally “readable” notes compared with physician-created notes. However, since each individual note corresponded to a unique patient encounter, there was no true “gold standard” for the information transmitted during that visit. This highlights the power of using simulation as an objective tool for determining competency, by controlling for the actual clinical content verbalized. Given the variability among scribe training and experience, their ability is likely also variable. Through the use of high-fidelity simulation exercises, one can standardize their training to ensure that all scribes reach objective benchmarks required for clinical practice.

### Limitations

It is important to note some important limitations to this study. Whereas this study focused on note creation, which is the primary role of the scribe, it did not address other scribe-specific activities such as data entry and data gathering [22,23]. Although we have previously demonstrated feasibility in integrating this into EHR-focused simulations, examining these other tasks will need to be the focus of future studies. Second, this study was a proof-of-concept study with a small number of scribes in a single specialty. Whereas the differences in note content and structure were noteworthy, a much larger cohort will be required to fully define the magnitude and scope of any potential safety issues in documentation and EHR usage. This is even more important, given the wide spectrum in baseline scribe training and prior experience in medical care before functioning as a scribe. Third, even though the simulations were designed to be easily deployed across multiple environments, additional studies will be required to determine the quantity and content of training required for novice educators (eg, providers) to access, deploy, and assess the work output from these activities, especially in community and rural settings. Finally, in real-world workflow, scribe notes must be attested and signed by an attending physician. Thus, it is unclear how much of the variance observed in the note structure would persist in actual clinical care after this final, attending physician–level vetting.

### Conclusions

In conclusion, our study highlights the variability of scribe documentation and the need for a more standardized approach to training. This proof-of-concept study demonstrated a means of effectively evaluating scribe performance.

## Abbreviations

CWH: Center for Women’s Health
EHR: electronic health record
HIPAA: Health Insurance Portability and Accountability Act
HITECH: Health Technology for Economic and Clinical Health
NCATS: National Center for Advancing Translational Sciences
NIH: National Institutes of Health
Ob-Gyn: Obstetrics and Gynecology
OCTRI: Oregon Clinical and Translational Research Institute
OHSU: Oregon Health & Science University
PPV: positive predictive value
